# Modeling larval malaria vector habitat locations using landscape features and cumulative precipitation measures

**DOI:** 10.1186/1476-072X-13-17

**Published:** 2014-06-06

**Authors:** Robert S McCann, Joseph P Messina, David W MacFarlane, M Nabie Bayoh, John M Vulule, John E Gimnig, Edward D Walker

**Affiliations:** 1 Department of Entomology, Michigan State University, East Lansing, MI, USA; 2 Department of Geography, Michigan State University, East Lansing, MI, USA; 3 Department of Forestry, Michigan State University, East Lansing, MI, USA; 4 Centre for Global Health Research, Kenya Medical Research Institute/Centers for Disease Control and Prevention, Kisumu, Kenya; 5 Division of Parasitic Diseases and Malaria, Centers for Disease Control and Prevention, Atlanta, GA, USA; 6 Department of Microbiology and Molecular Genetics, Michigan State University, East Lansing, MI, USA; 7 Current address: Laboratory of Entomology, Wageningen University and Research Centre, PO Box 8031, Wageningen 6700 EH, Netherlands

**Keywords:** Random forest, Logistic regression, *Anopheles gambiae*, Larval habitats, Predictive models

## Abstract

**Background:**

Predictive models of malaria vector larval habitat locations may provide a basis for understanding the spatial determinants of malaria transmission.

**Methods:**

We used four landscape variables (topographic wetness index [TWI], soil type, land use-land cover, and distance to stream) and accumulated precipitation to model larval habitat locations in a region of western Kenya through two methods: logistic regression and random forest. Additionally, we used two separate data sets to account for variation in habitat locations across space and over time.

**Results:**

Larval habitats were more likely to be present in locations with a lower slope to contributing area ratio (i.e. TWI), closer to streams, with agricultural land use relative to nonagricultural land use, and in friable clay/sandy clay loam soil and firm, silty clay/clay soil relative to friable clay soil. The probability of larval habitat presence increased with increasing accumulated precipitation. The random forest models were more accurate than the logistic regression models, especially when accumulated precipitation was included to account for seasonal differences in precipitation. The most accurate models for the two data sets had area under the curve (AUC) values of 0.864 and 0.871, respectively. TWI, distance to the nearest stream, and precipitation had the greatest mean decrease in Gini impurity criteria in these models.

**Conclusions:**

This study demonstrates the usefulness of random forest models for larval malaria vector habitat modeling. TWI and distance to the nearest stream were the two most important landscape variables in these models. Including accumulated precipitation in our models improved the accuracy of larval habitat location predictions by accounting for seasonal variation in the precipitation. Finally, the sampling strategy employed here for model parameterization could serve as a framework for creating predictive larval habitat models to assist in larval control efforts.

## Introduction

Malaria is one of the most significant infectious diseases affecting people in poverty, with an estimated 219 million cases of malaria worldwide in 2010 killing 660,000 people [1]. An estimated 1.44 billion people in South America, Africa, and Asia lived in areas with stable transmission of malaria caused by *Plasmodium falciparum* (Welch), yet the risk of *P. falciparum* transmission varies considerably across its range [2]. Even at fine scales, the spatial distribution of malaria is heterogeneous, differing among households within a community [3-6]. Of course, socioeconomic and immunological differences contribute to the spatial heterogeneity of malaria [3,4]. Additionally, landscape factors contribute to the spatial distribution of malaria [5,7], which is likely an indirect relationship ultimately due, in part, to the influence of landscape factors on the locations of the aquatic habitats of the vector mosquito larvae. The spatial distribution of the larval habitats partially determines the spatial distribution of the adult malaria vectors in many landscapes [5,8-10]. Subsequently, the heterogeneous spatial distribution of malaria vectors among households coincides with the spatial distribution of malaria parasitemia in some landscapes [5]. Therefore, understanding the factors that determine the distribution of the larval habitats facilitates our understanding of the spatial determinants of malaria transmission.

The vast majority of deaths from malaria (91%) occur in Africa [1], where the primary vector mosquitoes are among the most efficient vectors of malaria in the world. Two of the most widely distributed vectors in Africa are *Anopheles gambiae* s.s. Giles and *Anopheles arabiensis* Patton, which are both members of a species complex of eight closely related, morphologically indistinguishable species known collectively as *Anopheles gambiae* s.l. [11]. In many regions the larval habitats of *An. gambiae* s.s. and *An. arabiensis* are similar, and in fact, the two species are often found within the same larval habitats [12-14]. These larval habitats are generally smaller, temporary bodies of standing water persisting for about 20 to 40 days [12,15], with rain being the main source of the water.

The locations of larval *An. gambiae* s.l. habitats are associated with certain environmental features of landscapes. Previous studies have found more larval habitats closer to streams [16] and in locations with agricultural land uses [16,17]. Others have used a topographic wetness index (TWI) [18] to predict the locations of larval habitats, finding more larval habitats in locations having a combination of greater upslope area contributing to drainage and less slope [19-21]. The influence of soil types on the presence of larval habitats has largely been ignored, although Bøgh and colleagues [22] found larval habitats exclusively in alluvial soils in The Gambia. Finally, seasonal differences in rainfall likely influence the number of larval habitats on the landscape [12,16,19,20,23].

The objectives of this study were to create a model for predicting larval *An. gambiae* s.l. habitat locations using landscape variables that predict the likelihood of standing water bodies, and to account for seasonal changes in habitat probability based on accumulated precipitation. A model for accurately predicting the locations of malaria vector larval habitats has multiple utilities. First, it allowed us to investigate the links between larval habitat distribution and adult malaria vector distribution across a large landscape where manually mapping the larval habitats is infeasible (McCann et al. *in preparation*). Additionally, such a model could be useful for malaria control programs, allowing program managers to focus their efforts to areas where larval habitats are most likely to occur.

### Study site

**Figure 1:**
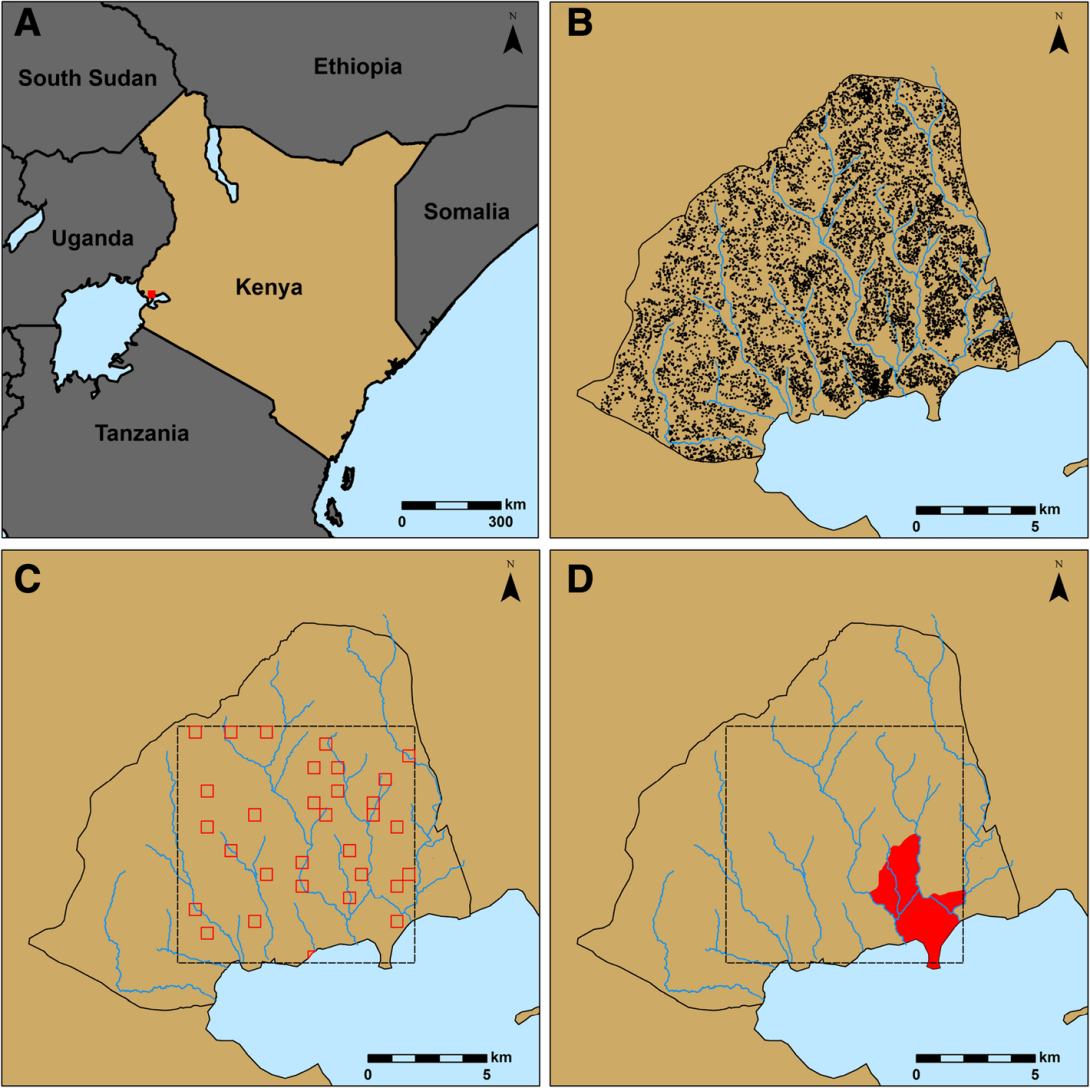
**Study site and survey locations. (A)** Map of Kenya with red square indicating location of the study region in western Kenya. **(B)** Map showing the boundaries of Asembo and the streams within the community, with black dots representing all households. **(C)** 10 by 10 km study site shown as dashed black line with thirty-one 500 by 500 m quadrats surveyed for larval habitats shown as red boxes. **(D)** Location of the neighboring villages of Aduoyo-Miyare and Nguka, sites of the 15 monthly ground surveys for larval habitats, shown in red.

The Asembo region of Rarieda District in western Kenya (Figure 1A) is a rural community of about 60,000 people covering about 200 km^2^. Most of the residents are subsistence farmers, and the landscape is largely dominated by small-scale agriculture. Small plots of land generally surround family-based groups of houses, or compounds, further arranged into villages. While the compounds are highly dispersed within villages, the boundaries between villages are often discernable only by residents [24] (Figure 1B). Asembo sits in the lowlands along the shores of Lake Victoria, with elevations ranging from 1,100 m to 1,400 m above sea level and low topographic relief. Networks of streams run across the region and drain into Lake Victoria. Farmland is common in these low-lying drainage basins, as well as throughout the region. Houses are mostly absent within 100 m of the streams. Rainfall is seasonally bimodal but may occur year round, with monthly precipitation totals ranging from 7 to 490 mm and yearly totals ranging from 1,100 to 1,800 mm from 2003 through 2012.

Malaria is holoendemic in Asembo, with parasitemia rates in children under 5 being around 50% in 2009 [25]. Similar to rainfall patterns, malaria transmission occurs year round, with seasonal peaks in May-July and October-November. The predominant species of malaria is *P. falciparum*. Two of the primary malaria vectors in the region are *An. gambiae* s.s. and *An. arabiensis*, the only two members of the *An. gambiae* s.l. species complex found here. The other primary malaria vector in the region is *Anopheles funestus* Giles. However, *An. funestus* and *An. gambiae* s.l. larvae do not generally occupy the same habitats, as the larval habitats of *An. funestus* are generally larger and more permanent than those of *An. gambiae* s.l. [12]. Larval *An. gambiae* s.l. habitats are numerous and widespread in Asembo yet heterogeneously distributed [16]. This makes it difficult to establish a relationship between larval habitats and the spatial distribution of the adult vectors or malaria prevalence in people. A 10 by 10 km study site was defined within Asembo to examine variation in the determinants of larval habitat location across a relatively large area. Because we wanted to include the lakeshore in the study site, the southern border fell largely within the lake, leaving 96.43 km^2^ of actual landmass in the 10 by 10 km site.

## Methods

### Larval habitat ground surveys

The 10 by 10 km study site was divided into 500 by 500 m quadrats for larval *An. gambiae* s.l. habitat ground surveys. After excluding the quadrats that fell completely in the lake, we selected quadrats for larval habitat ground surveys using spatially stratified random sampling from the remaining 393 quadrats (Figure 1C). For spatial stratification the 10 by 10 km area was divided into 2 by 2 km blocks. The 500 by 500 m quadrats were randomly selected from groups defined by the 2 by 2 km blocks. Spatial stratification was implemented to avoid the problem of sampling a cluster of quadrats in a certain area of the grid, assuring spatial variation in the predictor variables [26]. The time required for surveying a quadrat varied greatly according to the number of larval habitats in the quadrat, which was not known *a priori*. Therefore, we surveyed as many quadrats as possible during the targeted time frame, which was the end of the long rainy season to coincide with the peak *An. gambiae* s.l. population level [27-29]. Thus, 31 quadrats were surveyed exhaustively over 22 days between 17 May 2011 and 4 July 2011.

All potential larval *An. gambiae* s.l. habitats found in the quadrats were georeferenced with GPS units. Six field workers spaced 20 m from each other walked from one end of a quadrat to the other, using ArcPad (ESRI, Redlands, CA) on a GPS unit for navigating the borders of the quadrat. This was repeated until the entire quadrat was covered, usually in four to five passes. This approach allowed us to say, with certainty, where habitats were absent during the survey. In addition to recording the locations of each larval habitat, we recorded the presence or absence of *Anopheles* larvae. Larval *An. gambiae* s.l. habitats were defined as any standing body of water, regardless of whether *Anopheles* were present on the day of the ground survey, and falling under the following categories: drainage channel, burrow pit, rain pool, runoff, cluster of hoof prints, stream bed pool, pond/reservoir, wet meadow, well and tire track [15]. For a subset of habitats (the first five *Anopheles-*positive habitats for each of the four to five passes across each quadrat), *Anopheles* larvae and pupae were collected to confirm that the habitats were being used by *An. gambiae* s.l. All visible *Anopheles* larvae, up to a maximum of 20, were collected using a 300 ml dipper or plastic pipette as appropriate according to the size of the habitat. The specimens were transported to the lab for species identification. Larvae were raised to fourth-stage instars for identification, while pupae were allowed to eclose as adults before identification. All identifications were done according to Gillies and Coetzee [30].

To capture variation in habitat location across time due to seasonal rainfall patterns, additional ground surveys were conducted monthly in two neighboring villages, Aduoyo-Miyare and Nguka, covering 6.22 km^2^ within the 10 by 10 km study site (Figure 1D). Two local field workers with extensive knowledge of the villages walked throughout the whole of each village over the course of one to three days, depending on the number of habitats encountered, each month from April 2011 through June 2012. Potential *An. gambiae* s.l. habitats were defined and recorded as above. Thus, we had the ability to say where habitats were present and absent within the two villages each month.

### Environmental data

Spatial data for soils, land use-land cover (LULC), distance to the nearest stream, and TWI were created across the study site. These data were assembled in ArcGIS 10.0 (ESRI, Redlands, CA) in raster data structures with a spatial resolution of 20 m. All four datasets were treated as constant over time. Soil data were taken from the 1:1,000,000 exploratory soil map of Kenya, compiled by the Kenya Soil Survey in 1980 [31]. The three soil types in Asembo were 1) friable clay, 2) friable clay/sandy clay loam, and 3) firm, silty clay/clay. Of these soil types, friable clay drains more quickly, and firm, silty clay/clay drains more slowly. A satellite image from the IKONOS-2 sensor was used to create the LULC classification. Briefly, unsupervised classification was done using the K-means method [32] in ENVI 4.8 (Exelis Visual Information Solutions, Boulder, CO). Classes were combined into a binary data layer of agricultural or non-agricultural land use. All streams in Asembo were mapped using GPS units, and the Euclidean distance in meters to the nearest stream was calculated.

The TWI data were derived from a digital elevation model (DEM) of the study site. The DEM was created using local universal kriging to interpolate 11,130 GPS elevation records previously taken within Asembo [33,34]. The ArcGIS extension TauDEM 5.0 (Tarboton, Utah State University) was used to calculate a TWI. First, slope and flow direction were calculated using the deterministic infinite-node algorithm recommended by Tarboton, which is robust yet easily implemented [35]. Contributing area catchments were calculated using the flow direction and slope data. Finally, TWI was calculated as the ratio of the slope to the contributing area, and the values were rescaled to the range by taking:

$$\begin{array}{l} \left(\mathrm{TW}I_{i}–\min\left(\mathrm{TWI}\right)\right)\\ \;\times 100/\left(\max\left(\mathrm{TWI}\right)–\min\left(\mathrm{TWI}\right)\right)\;\mathrm{for}\;\mathrm{each}\;i\;\mathrm{value}\;\mathrm{of}\;\mathrm{TWI}. \end{array}$$

Because the TWI was calculated as the ratio of slope to contributing area, the lowest value (0) represented the wettest locations, while the highest TWI value (100) represented the driest areas.

**Figure 2:**
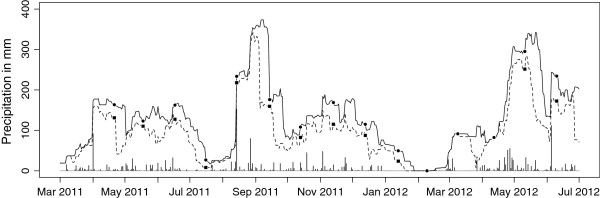
**Precipitation data from the Kisumu airport for March 2011 through July 2012.** Each point along the x-axis represents one day. The vertical bars show the daily precipitation total. The solid line shows the cumulative 30-day precipitation total (i.e. the level of the line for each day represents the summation of precipitation totals over the previous 30 days), which was used in the logistic regression model for the 15-month dataset. Similarly, the dashed line shows the daily fluctuation of the cumulative 21-day total used in the random forest model of the same data. Circles and squares show the last day of ground surveys for each month in Aduoyo-Miyare and Nguka. Kisumu airport is about 40 km east of Asembo.

**Figure 3:**
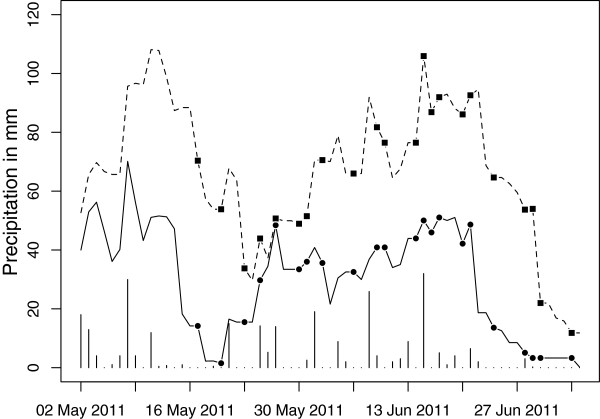
**Precipitation data from the Kisumu airport for 2 May 2011 to 4 July 2012.** Each point along the x-axis represents one day. The vertical bars show the daily precipitation total. The solid line shows the cumulative 6-day precipitation total (i.e. the level of the line for each day represents the summation of precipitation totals over the previous 6 days), which was used in the logistic regression model of the 10 by 10 km dataset. Similarly, the dashed line shows the daily fluctuation of the cumulative 14-day precipitation total used in the random forest model of the same data. Squares and circles show the days of the ground surveys in the 31 quadrats. Kisumu airport is about 40 km east of Asembo.

Daily precipitation totals for March 2011 to July 2012, as measured by the weather station at the Kisumu Airport (about 40 km east of Asembo), were downloaded from the National Climatic Data Center’s Global Summary of Day (GSoD) database (Figures 2 and 3). For missing daily data at the Kisumu weather station (n = 19 of 489 days), the inverse distance weighted mean of surrounding GSoD weather stations (within 250 km) was used. Cumulative *n*-day precipitation totals were calculated by summing the precipitation total of a given day with the previous *n* days for *n* = 0 to 30 days. Each *n*-day precipitation total had a daily temporal resolution, treated as spatially constant across our study site. From the resulting 31 measures of cumulative precipitation, we selected the best cumulative *n*-day total for each model based on the criteria outlined below.

### Statistical methods

We used two approaches for modeling the distribution of larval habitats across the landscape, logistic regression and *random forest*[36]. Logistic regression is commonly used in species distribution modeling [20,21,37]. Ecologists have recently started using the random forest method as well, because it does not require any assumptions about the distribution of the data [38,39]. Random forest is a machine learning classification method that extends classification and regression tree (CART) approaches, which work by recursive binary partitioning of the data space into increasingly homogenous regions [39,40]. Random forest works by fitting and combining many CARTs to create a more accurate prediction [36,39].

Both methods were used separately on the two datasets (one from the 10 by 10 km area and the other from the 15 monthly ground surveys in Aduoyo-Miyare and Nguka). For both methods, the unit of analysis was a 20 m pixel. Because each of the 31 quadrats in the 10 by 10 km area was surveyed once, each pixel in that dataset had a single value for all of the variables described above (habitat presence/absence, TWI, soil, LULC, distance to stream, and 31 values of *n*-day precipitation). The value of *n*-day precipitation was based on the day a given quadrat was surveyed (Figure 3). In the Aduoyo-Miyare and Nguka dataset, each pixel was repeated 15 times. The value of *n*-day precipitation was based on the final day of ground surveys, which took only 1 to 3 days, each month (Figure 2). Habitat presence/absence was determined by the ground survey data for each month, while TWI, soil, LULC and distance to stream were constant for a given pixel across all 15 months.

We built a series of candidate logistic regression models to select the most useful predictor variables. To determine which *n*-day precipitation measure to use, each cumulative precipitation measure was used alone as the predictor variable in separate regression models. Of these, the model with the lowest BIC determined the cumulative precipitation measure used in the subsequent logistic regression candidate models. The five predictor variables (TWI, soil, LULC, distance to stream, and precipitation) were then used in all 31 possible combinations to build candidate logistic regression models for each of the two datasets. To restrict the candidate model sets to relatively simple models with easily interpretable parameters, interactions among the five predictor variables were not included. The top models were again selected according to the lowest BIC. While the locations of larval *An. gambiae* s.l. habitats are generally clustered, we did not account for spatial autocorrelation in the logistic regression models presented here. Previous studies modeling larval habitats have found similar results for logistic regression models with and without parameters accounting for spatial autocorrelation [19-21]. The logistic regression models were implemented in the statistical software R 2.14.2 (R Development Core Team, Vienna, Austria).

We implemented the random forest approach using the R package ‘randomForest’ [41]. The best cumulative precipitation measure for use in the random forest models was determined by the mean decrease in the Gini impurity criterion when removing the variable from the full model (TWI, soil, LULC, distance to stream, and precipitation). The cumulative precipitation measure with the highest mean decrease in the Gini impurity criterion was used in the final random forest models. Gini impurity criteria were also used to measure variable importance in the random forest models. A greater mean decrease in the Gini impurity criterion suggests a stronger association with the response variable [36].

The top models from both approaches within each dataset were evaluated by determining their accuracy at predicting larval habitat presence and absence for holdout data. Fifty percent of each dataset was randomly selected as a holdout dataset before model building. Evaluation of model accuracy required the selection of a threshold at which to convert predicted probabilities into larval habitat presence or absence. Because threshold specific accuracy statistics can be sensitive to the threshold used for conversion, we generated an optimal threshold value by minimizing the absolute value of the difference between sensitivity and specificity [42]. This approach was chosen because both sensitivity and specificity were equally important for the intended application of the predictive models. To assess the performance of each model, we calculated the sensitivity, specificity, percent correctly classified (PCC), and kappa of each approach at each of the thresholds from the methods above. We also calculated the threshold-independent area under the curve (AUC) statistic [43] from receiver operating characteristics (ROC) plots using the R package ‘SDMTools’ [44].

Finally, we calculated Pearson’s correlation coefficient among the cumulative precipitation measures to assess differences among the temporal-resolution/modeling-approach combinations. To quantify the contribution of cumulative 30-day precipitation to variation in the number of habitats found each month in Aduoyo-Miyare and Nguka, we used simple linear regression.

## Results

In the 31 sampling quadrats selected from the 10 by 10 km study site, we recorded the locations of 1,673 larval *An. gambiae* s.l. habitats. Six of the quadrats did not have any larval habitats, while the mean number of habitats per 500 by 500 m quadrat was 54. *Anopheles* larvae were present in 921 of the 1,673 habitats on the day each habitat was recorded. As detailed in the methods, *Anopheles* larvae and pupae were collected from 141 of the habitats, 77% of which were occupied by *An. gambiae* s.l. on the day of collection. Most of the larvae and pupae (79%) were identified as *An. gambiae* s.l. The other species collected were *An. funestus* (1.1%), *Anopheles coustani* Laveran (6.7%), *Anopheles rufipes* (Gough) (5.3%), *Anopheles maculipalpis* Giles (2.5%) and *Anopheles pharoensis/squamosus* Theobald (3.9%).

**Figure 4:**
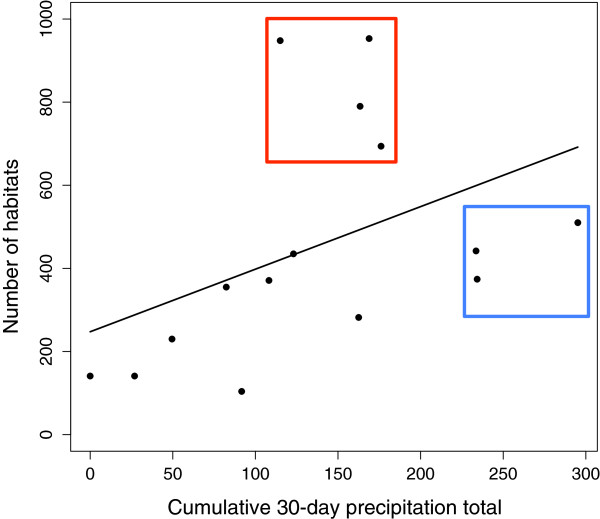
**Number of larval habitats by cumulative precipitation.** Scatter plot of the number of habitats recorded in Aduoyo-Miyare and Nguka each month (n = 15) by the cumulative 30-day precipitation for the last day of ground surveys for that month. Line shows prediction of linear regression, R^2^ = 0.1931, p = 0.1012. The red and blue boxes highlight variation in the residual error discussed further in the text.

In 15 monthly ground surveys in Aduoyo-Miyare and Nguka, a total of 6,770 larval *An. gambiae* s.l. habitats were recorded. The number of larval habitats in this area varied by month, ranging from 104 to 953 with a mean of 451. The number of larval habitats recorded in the two villages each month increased with increasing cumulative 30-day precipitation on the final day of ground surveys each month, though considerable variation was observed (R^2^ = 0.1931, p = 0.1012; Figure 4).

The best cumulative precipitation total to use in the models differed between the datasets and between the modeling approaches. For the 15 monthly ground surveys in Aduoyo-Miyare and Nguka, the logistic regression model for 30-day cumulative precipitation had the lowest BIC within the precipitation candidate models (Figure 2), whereas the random forest model using the cumulative 21-day precipitation (Figure 2) had the highest mean decrease in the Gini impurity criterion. For the 10 by 10 km data, the logistic regression model for 6-day cumulative precipitation had the lowest BIC within the precipitation candidate models (Figure 3), while the random forest model using the cumulative 14-day precipitation (Figure 3) had the highest mean decrease in the Gini impurity criterion. Each of these precipitation measures was used within its respective dataset and modeling approach moving forward. However, it should be noted that these cumulative precipitation totals were moderately to highly correlated with each other (Table 1).

**Table 1:** Pearson’s correlation coefficient (r) matrix of cumulative precipitation measures

	**0-day**	**6-day**	**14-day**	**21-day**	**30-day**
A) 17 May - 4 July 2011 (temporal scale for 10 by 10 km dataset)					
6-Day	0.411	1.000	0.721	0.477	0.611
14-Day	0.332	0.721	1.000	0.794	0.578
B) April 2011 - June 2012 (temporal scale for Aduoyo-Miyare and Nguka dataset)					
21-Day	0.408	0.555	0.841	1.000	0.979
30-Day	0.336	0.537	0.819	0.979	1.000

(A) For the 22 days of larval habitat ground surveys in the 10 by 10 km area from 17 May to 4 July 2011, and (B) For the last day of larval habitat ground surveys each month in Aduoyo-Miyare and Nguka from April 2011 to June 2012. 0-day refers to precipitation total for the day of ground surveys, while 6-day, 14-day, 21-day, and 30-day refer to the cumulative precipitation total for the day of the ground surveys plus the previous 6 days, 14 days, 21 days, or 30 days, respectively.

The environmental variables used in the best logistic regression models for predicting the locations of larval *An. gambiae* s.l. habitats differed slightly between the datasets. For the 15-month dataset from Aduoyo-Miyare and Nguka, the logistic regression model with the lowest BIC included all five of the variables (Table 2). No other model had a ΔBIC less than 20. Larval habitats were more likely to be found in locations with a lower TWI (i.e. wetter because of a lower slope to contributing area ratio), closer to streams, in agricultural land use, and in the friable clay/sandy clay loam soil type (Table 3). The probability of a larval habitat increased with increasing cumulative 30-day precipitation (Table 3).

**Table 2:** Top four logistic regression candidate models for the 15 monthly ground surveys in Aduoyo-Miyare and Nguka

**Model**	**BIC**	**ΔBIC**	***w***
TWI + LULC + DS + Soil + Precip.	45933.1	NA	0.9999
TWI + DS + Soil + Precip.	45955.6	22.5	<0.001
TWI + LULC + DS + Precip.	46045.5	112.3	<0.001
TWI + DS + Precip.	46073.7	140.5	<0.001

Based on Bayesian information criterion (BIC), in order of increasing difference in BIC from the top model (ΔBIC) and decreasing BIC weight, *w*. TWI, topographic wetness index; LULC, land use-land cover; DS, distance to the nearest stream; Precip., cumulative 30-day precipitation total; NA, not applicable.

**Table 3:** Odds ratios for top logistic regression model from the Aduoyo-Miyare and Nguka data (15 monthly surveys)

	**Odds ratio**	**95% lower**	**95% upper**
(Intercept)	0.0378	0.0334	0.0426
TWI	0.9365	0.9324	0.9405
LULC, Ag:NonAg	1.3371	1.2096	1.4780
DS	0.9980	0.9978	0.9981
Soil, Type3:Type2	0.7127	0.6715	0.7564
Precip.	1.0033	1.0029	1.0036

Odds ratios are presented with 95% CI. TWI, topographic wetness index; LULC, land use-land cover; DS, distance to the nearest stream; Precip., cumulative 30-day precipitation total; Ag:NonAg, odds ratio of agricultural to nonagricultural LULC; Type3:Type2, odds ratio of the firm, silty clay/clay soil type to the friable clay/sandy clay loam soil type.

For the 10 by 10 km dataset, the logistic regression model with the lowest BIC included four of the variables (TWI, distance to stream, soil type, and cumulative 6-day precipitation). No other model had a ΔBIC less than 9 (Table 4). Larval habitats were again more likely to be found in locations with a lower TWI, closer to streams, and in the friable clay/sandy clay loam soil type (Table 5). Counterintuitively, the probability of a larval habitat according to this model decreased with increasing cumulative 6-day precipitation.

**Table 4:** The top five logistic regression candidate models for the 10 by 10 km area

**Model**	**BIC**	**ΔBIC**	***w***
TWI + DS + Soil + Precip.	6482.6	0	0.9822
TWI + LULC + DS + Soil + Precip.	6491.8	9.3	0.0096
TWI + DS + Precip.	6492.2	9.6	0.0082
TWI + LULC + DS + Precip.	6502.0	19.4	< 0.001
TWI + DS + Soil	6658.0	175.4	< 0.001

Based on Bayesian information criterion (BIC), in order of increasing difference in BIC from the top model (ΔBIC) and decreasing BIC weight, *w*. TWI, topographic wetness index; LULC, land use-land cover; DS, distance to the nearest stream; Precip., cumulative 30-day precipitation total.

**Table 5:** Odds ratios for the top logistic regression model for the 10 by 10 km area data

	**Odds ratio**	**95% lower**	**95% upper**
(Intercept)	0.3156	0.2472	0.4030
TWI	0.9117	0.8988	0.9248
DS	0.9973	0.9969	0.9977
Soil, Type2:Type1	1.9105	1.4994	2.4343
Soil, Type3:Type1	1.4970	1.2024	1.8639
Precip.	0.9700	0.9656	0.9745

Odds ratios are presented with 95% CI. TWI, topographic wetness index; DS, distance to the nearest stream; Precip., cumulative 6-day precipitation total; Type2:Type1, odds ratio of the friable clay/sandy clay loam soil type to the friable clay soil type; Type3:Type1, odds ratio of the firm, silty clay/clay soil type to the friable clay soil type.

**Figure 5:**
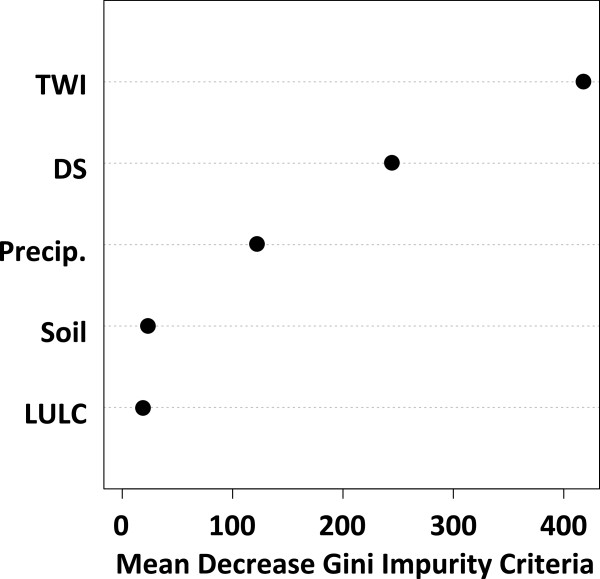
**Mean decrease in the Gini impurity criterion as a measure of variable importance.** Shown for the random forest model in the 10 by 10 km dataset. Greater decreases indicate a stronger association with the response. TWI, topographic wetness index; LULC, land use-land cover; DS, distance to the nearest stream; Precip., cumulative 14-day precipitation total.

**Figure 6:**
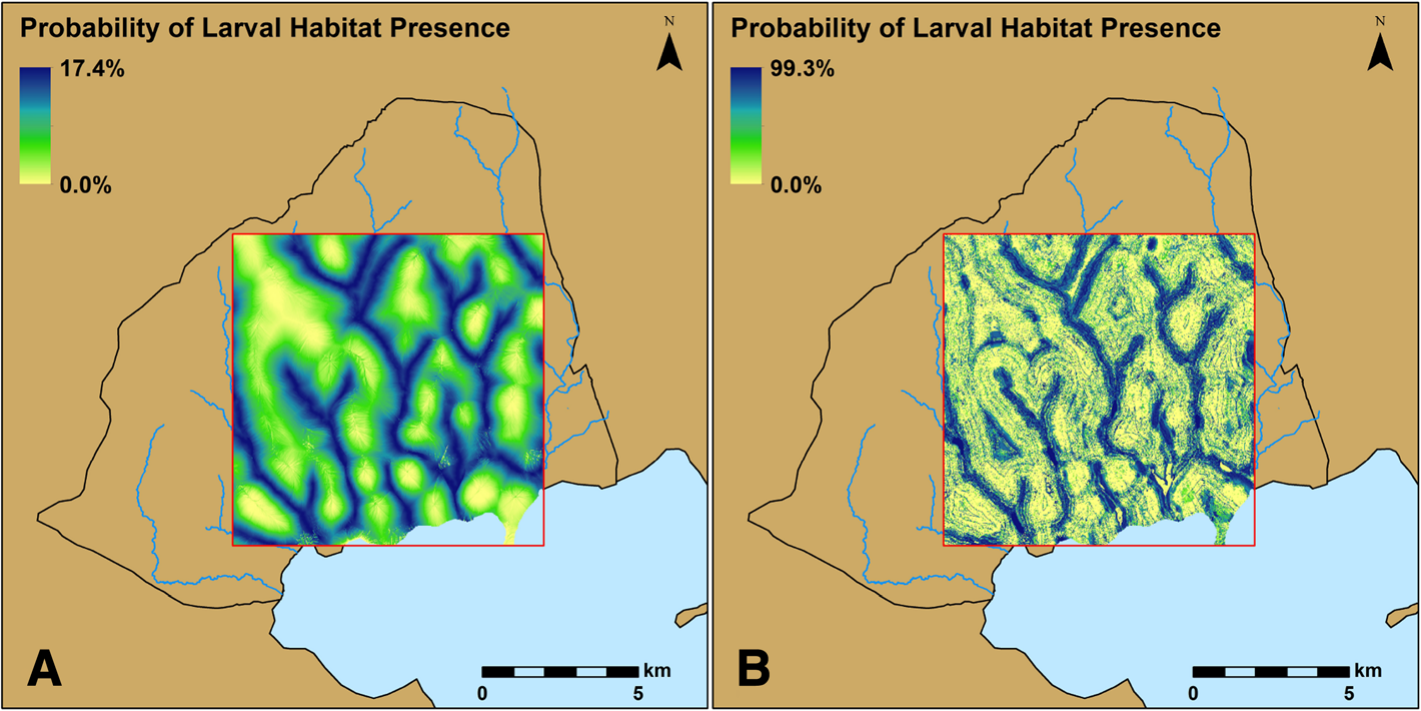
**Probability of larval habitat presence across the 10 by 10 km study site.** As predicted by **(A)** the logistic regression model using TWI, distance to stream and soil type; and **(B)** the random forest model using TWI, distance to stream, soil type and LULC.

The most accurate model for predicting larval *An. gambiae* s.l. habitat locations in the 10 by 10 km area was the random forest method with all five variables (AUC = 0.864). In this model, TWI had the greatest mean decrease in the Gini impurity criterion, followed by distance to stream, precipitation, soil and LULC (Figure 5). Removing the cumulative 14-day precipitation from this model reduced the accuracy of the model (Table 6). The best logistic regression model for the 10 by 10 km area was less accurate than the random forest model when evaluated against the holdout data (Table 6). When the probabilities of larval habitat location across the entire 10 by 10 km study site were estimated using the most accurate models from each method, the random forest model clearly produced a more heterogeneous landscape at a fine scale than that produced by the logistic regression method (Figure 6).

**Table 6:** Comparison of models predicting the presence of larval habitats in the 10 by 10 km area

**Model**	**AUC**	**Sensitivity**	**Specificity**	**PCC**	**Kappa**
RF: TWI + DS + Soil + LULC + Precip.	0.864	0.806	0.789	0.790	0.216
RF: TWI + DS + Soil + LULC	0.808	0.750	0.725	0.726	0.145
LR: TWI + DS + Soil + Precip.	0.799	0.748	0.709	0.711	0.133

Two random forest (RF) models are shown, with and without Precip. (the cumulative 14-day precipitation total). The best logistic regression (LR) model is also shown. TWI, topographic wetness index; LULC, land use-land cover; DS, distance to the nearest stream; AUC, area under the receiver operating curve; PCC, percent correctly classified.

The most accurate model for predicting larval *An. gambiae* s.l. habitat locations over the 15 monthly surveys in Aduoyo-Miyare and Nguka was the random forest method with all five variables (Table 7). As above, removing the cumulative 21-day precipitation from the model reduced its accuracy. The best logistic regression model for the 15 monthly surveys in Aduoyo-Miyare and Nguka was less accurate than the random forest model when evaluated against the holdout data (Table 7).

**Table 7:** Comparison of models predicting the presence of larval habitats in Aduoyo-Miyare and Nguka

**Method of optimizing**	**AUC**	**Sensitivity**	**Specificity**	**PCC**	**Kappa**
RF: TWI + DS + Soil + LULC + Precip.	0.871	0.820	0.773	0.774	0.102
RF: TWI + DS + Soil + LULC	0.827	0.659	0.936	0.930	0.268
LR: TWI + DS + Soil + Precip.	0.733	0.621	0.704	0.703	0.045

Two random forest (RF) models are shown with and without Precip. (the cumulative 14-day precipitation total). The best logistic regression (LR) model is also shown. TWI, topographic wetness index; LULC, land use-land cover; DS, distance to the nearest stream; AUC, area under the receiver operating curve; PCC, percent correctly classified.

## Discussion

The use of models to predict the distribution of species is common in ecology [37], and novel approaches to building these models such as random forest have become more widely available in recent years. We used two methods to predict the probability of larval *An. gambiae* s.l. habitat across the landscape and over time, and the random forest method produced more accurate models than the logistic regression method. This may be due to the differences in predicted heterogeneity of larval habitats at fine scales between the two methods, which can be compared across the entire 10 by 10 km study site (Figure 6). Predictions from the random forest model are more fragmented, showing a closer proximity of high-probability locations to low-probability locations, relative to the estimates from the logistic regression model. The general pattern is similar for the predictions of both models at broad scales (Figure 6). However, the fine scale heterogeneity in the random forest estimates more closely reflects the nature of actual larval habitat distribution on the ground, where larval *An. gambiae* s.l. habitats are distributed as many small patches rather than one continuous, large patch.

The most important landscape variables for predicting larval habitat presence in these models were TWI and distance to the nearest stream. In the 10 by 10 km random forest model, the mean decreases in the Gini impurity criteria of TWI and distance to the nearest stream were much larger than those of LULC and soil (Figure 5), indicating a stronger association with the prediction of habitat presence [36]. In general practice, high quality soil and LULC data can be difficult to acquire. Given limited resources, our data suggest it is possible to build reasonably accurate larval habitat models without these two landscape variables. Nonetheless, soil and LULC do show an association with habitat presence according to the logistic regression models presented here.

An important question in the application of predictive larval habitat models is whether models parameterized with data for habitat locations in one season are applicable to another season [20]. The creation of larval *An. gambiae* s.l. habitats (which are temporary, small bodies of standing water) depends on rainfall, which varies seasonally across the range of the species complex. One strategy to model differences between seasons is to account for variation in precipitation. In the random forest models, accumulated precipitation was less important than TWI and distance to the nearest stream, but it was a more important predictor variable than soil and LULC (Figure 5). Additionally, we found more larval habitats in months with more precipitation compared to the same area in months with less precipitation (Figure 4). Thus, including accumulated precipitation in our models improved the accuracy of larval habitat location predictions. These results should be interpreted with caution given the use of a single location about 40 km from the study site as the source of precipitation data. Daily precipitation totals can be spatially heterogeneous at that scale. Despite this limitation, it is clear from previous work that variation in precipitation influences larval *An. gambiae* s.l. habitats [12,16,19,20]. However, the relationship may be more complex than it first appears. For example, it may not be linear. Rather, the number of larval habitats may increase monotonically with accumulated precipitation up to a threshold, after which more of the water on the landscape flows as surface sheet or channeled water, which is unsuitable aquatic habitat for *An. gambiae* s.l. larvae. Additionally, different habitat types may respond differently to increasing accumulated precipitation. Standing water forming in drainage channels and stream bed pools may be described better by a threshold relationship than the water filling burrow pits, hoof prints and tire tracks, because the former develops from channel and sheet water made stationary by diminished water flows, whereas the latter forms from water accumulating into various catchments not associated with channels. These additional factors may explain some of the uneven residual error seen in Figure 4, where the 4 months in the red box falling above the fitted regression line have more larval habitats with a lower accumulated precipitation relative to the 3 months in the blue box falling below the fitted regression line.

The *n*-day cumulative precipitation measure used for each modeling approach within each dataset was selected according to the criteria outlined in the methods to maximize the predictive power of each model. However, comparing across modeling approaches within each dataset, the cumulative precipitation measures were highly correlated (Table 1). Thus, the choice between 21-day cumulative precipitation and 30-day cumulative precipitation, for example, may be less important in general practice than using either measure instead of the daily precipitation total (referred to as 0-day in Table 1). Comparing across datasets, which differed in temporal scale, the 6-day cumulative precipitation and the 30-day cumulative precipitation are only moderately correlated. Their differences in terms of model fit (BIC) could reflect temporal differences in hydrology on this landscape, but it may also reflect a limitation of the 10 by 10 km data collection (see below).A counterintuitive result of this study was that the odds of larval habitat presence decreased with increasing cumulative 6-day precipitation using the best logistic regression model of the 10 by 10 km data. Most likely this reflects a limitation of the 10 by 10 km data collection rather than the true influence of precipitation on larval habitat presence, given the range of cumulative 6-day precipitation over the 49-day period (1.5 mm – 51.1 mm; Figure 3). The sampling strategy for those data was designed to capture variation in landscape variables over space. While precipitation varied among the days of the ground surveys, we were not able to capture that variation over the full range of values for the landscape variables. Instead, the effect of accumulated precipitation in this particular model may be an indication of some other property differing between the quadrats sampled on days of higher and lower accumulated precipitation. Alternatively, the temporal scale over which larval habitats respond to variation in accumulated precipitation may be closer to monthly than daily. That is, ground surveys conducted at monthly intervals in the same area may be more likely to be different than daily samples within a month in the same area. As noted above, this may also reflect the use of a single location as the source of all precipitation data.

In addition to the use of precipitation data from one location, there were other limitations to this study. First, we did not account for spatial autocorrelation in the logistic regression models. Doing so may have slightly increased the confidence intervals associated with the parameters of those models, but it is unlikely to have changed the model comparisons or accuracy evaluations presented here. Previous studies modeling *An. gambiae* s.l. larval habitat locations have found similar results for logistic regression models with and without parameters accounting for spatial autocorrelation [19-21]. Second, there were additional variables we could have included in our analysis, such as a model-based wetness index (MWI) or normalized difference vegetation index (NDVI). MWI are similar to TWI, but MWI use simulations of distributed catchment models to account for differences between groundwater gradients and surface gradients, thereby creating more accurate topographic data [45]. We used TWI here because it has performed well in other models of *Anopheles* larval habitats [19-21], and is easily implemented compared with MWI. While our models using TWI showed high accuracy, further studies comparing the use of MWI and TWI in larval habitat modeling are needed. NDVI has also been associated with the distribution of malaria [46,47], although some studies have found contradicting results [17,48,49]. NDVI is an indirect measure of available moisture, but NDVI values are additionally influenced by vegetation type and phenology. Thus, we used accumulated precipitation as a measure of available moisture.

Finally, the models developed here exclusively used physical and environmental factors as predictor variables, but the formation of larval *An. gambiae* s.l. habitats also depends on human behavior. For example, landowners in Asembo create small drainage channels around fields. Standing water left behind in the channels creates habitats for *An. gambiae* s.l. larvae [15]. The locations of these drainage channels are often in low-lying agricultural areas, and therefore our models were able to predict the locations of most of the drainage channels. However, drainage channels are not found in 100% of low-lying agricultural areas, probably in part because of individual variation in landowner decision-making. Larval habitats formed from burrow pits and aggregations of hoof prints are also subject to variation in human behavior. While our models were able to correctly predict the locations of most of these habitats, interactions between the physical landscape and human behavior likely account for some of the locations identified incorrectly by the models.

The sampling designs of these two datasets allowed us to address two complementary goals. The monthly surveys in Aduoyo-Miyare and Nguka captured variation in precipitation across both dry and rainy seasons in the same landscape. This provided a stronger logical basis for inferences about the relationship between seasonal variation in precipitation and variation in the location and number of larval habitats. The small spatial extent of Aduoyo-Miyare and Nguka made monthly surveys more feasible, but it also limited the applicability of the model results across a larger area. Conversely, limiting the ground surveys of the 31 quadrats from the 10 by 10 km study site to one season likely impeded our ability to infer much about the effect of precipitation on these data. On the other hand, concentrating our sampling effort to increase replication across space in the 31 quadrats captured more variation in landscape variables, allowing us to apply the results of models based on these data to a larger area.

As a general application, the spatially stratified sampling strategy used in the 10 by 10 km site could serve as a framework for creating predictive larval habitat models for larval control. Targeted larval control is often cited as a useful application of predictive larval habitat models [20,21], and we agree that there is potential for this application. For example, malaria control programs could identify areas suited to environmental management such as filling in burrow pits and engineering drainage channels to drain more completely. Additionally, allowing larvicide application crews to focus on areas with a higher probability of larval habitat presence would reduce the time, and therefore the cost, of larviciding. However, models fitted to data from a single geographic location may have limited generalizability [50]. Malaria control programs could overcome this limitation by using spatially stratified random samples, repeated across a variable landscape, to build models that are useful over larger areas.

## Competing interests

The authors declare that they have no competing interests.

## Authors’ contributions

RSM, MNB, JMV, JEG and EDW designed the study and implemented the data collection. RSM, JPM and DWM analyzed the data and assessed the models. All authors participated in the preparation of the manuscript, and read and approved the final manuscript.
